# Publisher Correction: Flexible Learning-Free Segmentation and Reconstruction of Neural Volumes

**DOI:** 10.1038/s41598-018-36220-7

**Published:** 2018-11-29

**Authors:** Ali Shahbazi, Jeffery Kinnison, Rafael Vescovi, Ming Du, Robert Hill, Maximilian Joesch, Marc Takeno, Hongkui Zeng, Nuno Maçarico da Costa, Jaime Grutzendler, Narayanan Kasthuri, Walter J. Scheirer

**Affiliations:** 1 Department of Computer Science and Engineering, University of Notre Dame, Notre Dame, IN USA; 2 Center for Nanoscale Materials, Argonne National Laboratory, Lemont, IL USA; 3 Department of Neurobiology, University of Chicago, Chicago, IL USA; 4 Department of Materials Science and Engineering, Northwestern University, Evanston, IL USA; 5 Department of Neurology, Yale University, New Haven, CT USA; 6 Neuroethology Group, IST Austria, Klosterneuburg, Austria; 7 Allen Institute for Brain Science, Seattle, WA USA

Correction to: *Scientific Reports* 10.1038/s41598-018-32628-3, published online 24 September 2018

The incorrect Supplementary Information file was originally published with this Article. The correct Supplementary Information file now accompanies the Article.

