# Supplementary material for: Flexible Learning-Free Segmentation and Reconstruction of Neural Volumes
Source: Sci Rep. 2018 Sep 24;8:14247. doi: 10.1038/s41598-018-32628-3 (PMC6155135; doi:10.1038/s41598-018-32628-3)
Supplement: Supplementary file 1 — Supplementary Information [file 41598_2018_32628_MOESM1_ESM.pdf]

Supplementary Information: Flexible Learning-Free Segmentation and Reconstruction of Neural Volumes

Ali Shahbazi[1,+], Jeffery Kinnison[1,2,+], Rafael Vescovi[2,3], Ming Du[4], Robert Hill[5], Maximilian Joesch[6], Marc Takeno[7], Hongkui Zeng[7], Nuno Maçarico da Costa[7], Jaime Grutzendler[5], Narayanan Kasthuri[2,3], and Walter J. Scheirer[1,*]

[1]Department of Computer Science and Engineering, University of Notre Dame, Notre Dame, IN, USA
[2]Center for Nanoscale Materials, Argonne National Laboratory, Lemont, IL, USA
[3]Department of Neurobiology, University of Chicago, Chicago, IL, USA
[4]Department of Materials Science and Engineering, Northwestern University, Evanston, IL, USA
[5]Department of Neurology, Yale University, New Haven, CT, USA
[6]Neuroethology Group, IST Austria, Klosterneuburg, Austria
[7]Allen Institute for Brain Science, Seattle, WA, USA
[*]walter.scheirer@nd.edu
[+]These authors contributed equally to this work.

ABSTRACT

This document contains all supplemental figures and methods descriptions.

Supplementary Methods: Evaluation Metrics

In our experiments, we compared the SRB and two sSEM subvolume reconstructions to stacks of expert-annotated ground truth. Since 3D reconstruction is the goal of the work, we chose Hausdorff distance as an evaluation tool. It is a well-known evaluation method for 3D meshes and reconstructions[1]. Unlike other similarity scores (e.g., variation of information) that perform pixel-wise comparisons, the Hausdorff distance computes the maximum or mean minimum distance necessary to travel from any point in a set A to any point in a second set B. Consider a 3D reconstruction A and corresponding 3D ground-truth B: $A = \{a_1, a_2, ..., a_n\}$ and $B = \{b_1, b_2, ..., b_n\}$. The Hausdorff distance from A to B will be $\tilde{\delta_H}(A,B) = max_{a \in A} min_{b \in B} \|a - b\|$. In our formulation, the sets A and B consist of the 3D indices of positive pixels in the reconstructions and ground truth, respectively. Throughout this article, we use the convention A is in X/Y of the distance of B. Thus when we say A is in 1/1000 of the distance of B, this means the average distance between A and B is less than 0.001.

Supplementary Methods: U-Net & 3D U-Net Training

U-Net and 3D U-Net models were trained to segment cells in the SRB volume and APEX2-positive cells in the two sSEM subvolumes, the three volumes in this study for which expert manual annotations exist. For SRB, 80% of the dataset was used for training, with 10% held out for validation and 10% held out for testing. For the two sSEM subvolumes, a training set was drawn from a third disjoint subvolume of APEX-positive cells, 10% images of that training set were held out for validation, and the subvolumes themselves were used for testing.

For each volume, 20 randomly-initialized U-Nets were trained for 150 epochs on batches of 4 64×64 patches drawn from 80% of the volume in a random order, optimizing on binary cross-entropy loss with an SGD optimizer with a learning rate of 10^{-3} and momentum of .99, as in Ronneberger *et al.*[2]. We saved the weights that produced each architecture's best observed validation loss and used those weights to evaluate on the test set. 3D U-Net training followed the same pattern, training on batches of one $16 \times 32 \times 32$ block at a time. All training was carried out on NVIDIA Tesla K80 GPUs in the Visualization Cluster at the Argonne Leadership Computing Facility of Argonne National Laboratory.

Supplementary Methods: Non-Machine Learning Segmentation Method Evaluation

To make the FLoRIN pipeline as flexible as possible, it does not explicitly depend upon the NDNT algorithm to complete the Segmentation stage. To demonstrate the advantages of using NDNT over other learning-free segmentation methods, however,

we repeated the segmentation tasks using a variety of other thresholding methods[3–10] and morphological geodesic active contours (MGAC)[11]. All methods, including NDNT, were applied to all individual 2D images of each volume in this study. Additionally, the Otsu, Li, Yen, Triangle, and Isodata global thresholding methods were applied to each volume on individual 3D blocks of each volume at a time, using the same tiling methodology applied to NDNT, and to the entire 3D volume at once.

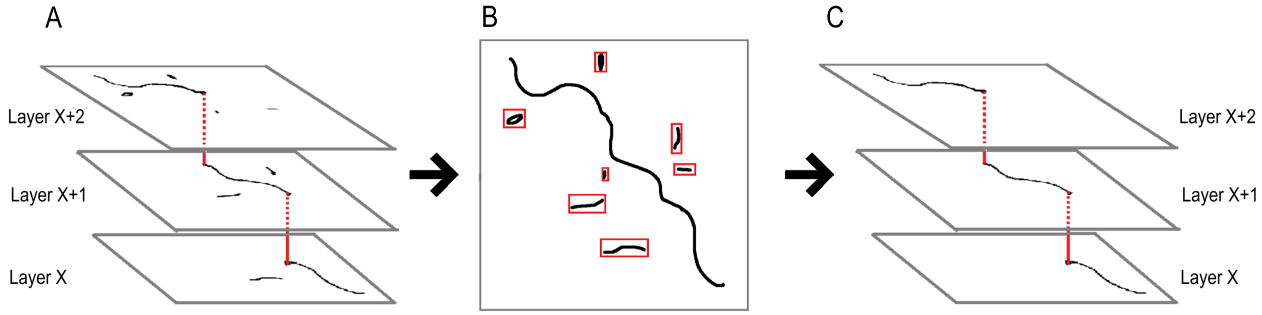

Supplementary Figure S 1. The 2D Identification process in FLoRIN first registers all connected components discovered in the Identification stage into a single plane. Connections are preserved by connecting components that overlap with one another in 2D, and unconnected components are discarded. The remaining components are then placed into a 3D volume.

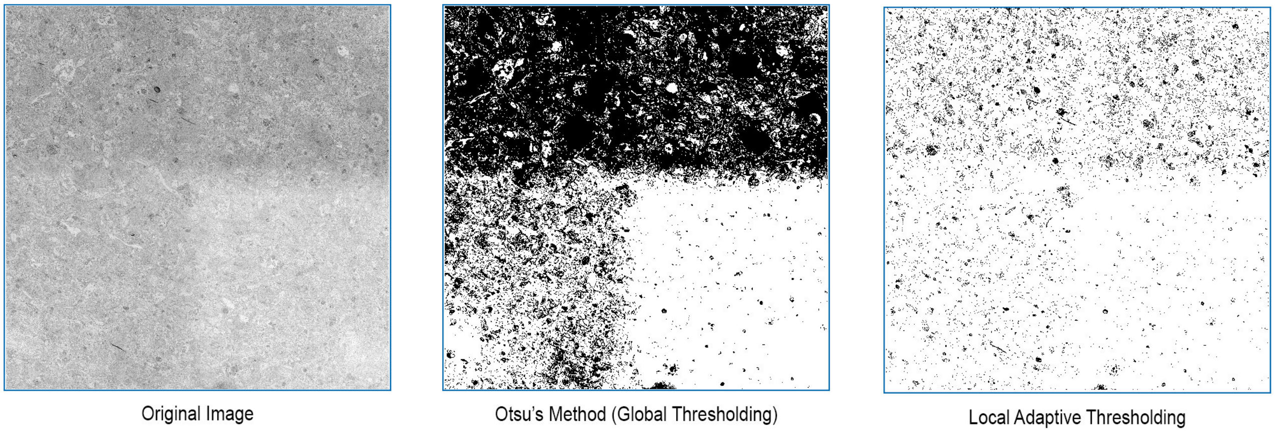

Supplementary Figure S 2. A comparison of the performance of global versus local thresholding methods on a noisy image. (Left) Neural images are often subject to large shifts in the grayscale distribution that can hamper reconstruction efforts. (Center) Otsu's method[5] binarizes images using a global threshold value, however due to grayscale shifts the binarization includes large portions of the image background. (Right) Our NDNT algorithm operates by observing a local neighborhood around each voxel to reduce the impact of distant noise. In this case, NDNT captures more of the features of interest despite large grayscale shifts.

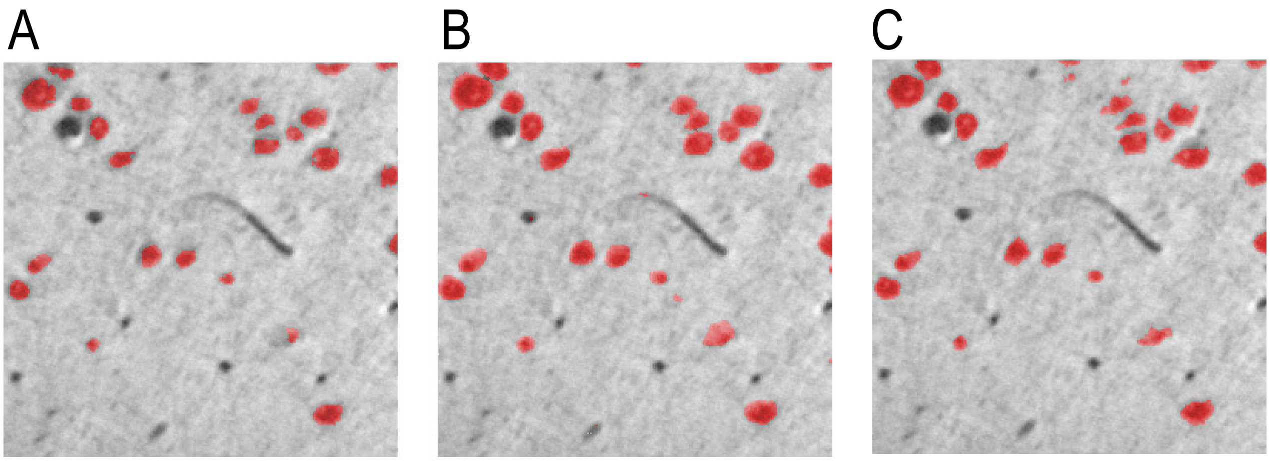

Supplementary Figure S 3. A comparison of SRB (A) manual annotations, (B) 3D U-Net cell segmentation, and (C) 3D FLoRIN cell segmentation. In general, 3D U-Net tends to over-segment the cells, incurring merge errors with nearby cells and on correctly identifying a vasculature segment as a cell. 3D FLoRIN, on the other hand, clearly separates grouped cells and does not misclassify vasculature. This image taken from the U-Net/3D U-Net training set.

Supplementary Table S 1. Running time comparison to the previous version of the pipeline[12].

Method	Running time
**Joesch et al. [12]**	136 min
**2D FLoRIN**	24.82 min
**3D U-Net**	3559.3 min

```
Data: img: an n-dimensional image to threshold
Data: n: the dimensionality of img
Data: d: an n-tuple containing the size of each dimension of img
Data: s: an n-tuple containing the dimensions of the box around each pixel
Data: t: the threshold value to use, number in range [0,1]
Result: binarization of img
let out be an array the same size as img;
intImg = img;
for i in 1..n do
    // Compute the cumulative summation over dimension i
    intImg = cumulativeSummation(intImg, i);
end
let indices be the set of all binary strings length n;
let low, hi, vertex be length n arrays filled with zeros;
parity = n mod 2;
foreach element e in intImg do
    x = index(intImg, e);
    for i in 1..n do
        low[i] = x[i] - s[i] / 2;
        hi[i] = x[i] - s[i] / 2;
        if low[i] < 1 then
            low[i] = 0;
        end
        if hi[i] > d[i] then
            hi[i] = d[i];
        end
    end
    count = ∏_{i=1}^{n} hi[i] − low[i];
    sum = 0;
    foreach idx in indices do
        p = 0;
        for i in 1..n do
            p = p + idx[i];
            if idx[i] = 1 then
                vertex[i] = hi[i];
            else
                vertex[i] = low[i];
            end
        end
        p = p mod 2;
        if p = parity then
            sum = sum + intImg[vertex];
        else
            sum = sum - intImg[vertex];
        end
    end
    if img[x] × count ≤ sum × (1.0 - t) then
        out[x] = 0;
    else
        out[x] = 1;
    end
end
return out
```

Supplementary Algorithm S 1: N-Dimensional Neighborhood Thresholding

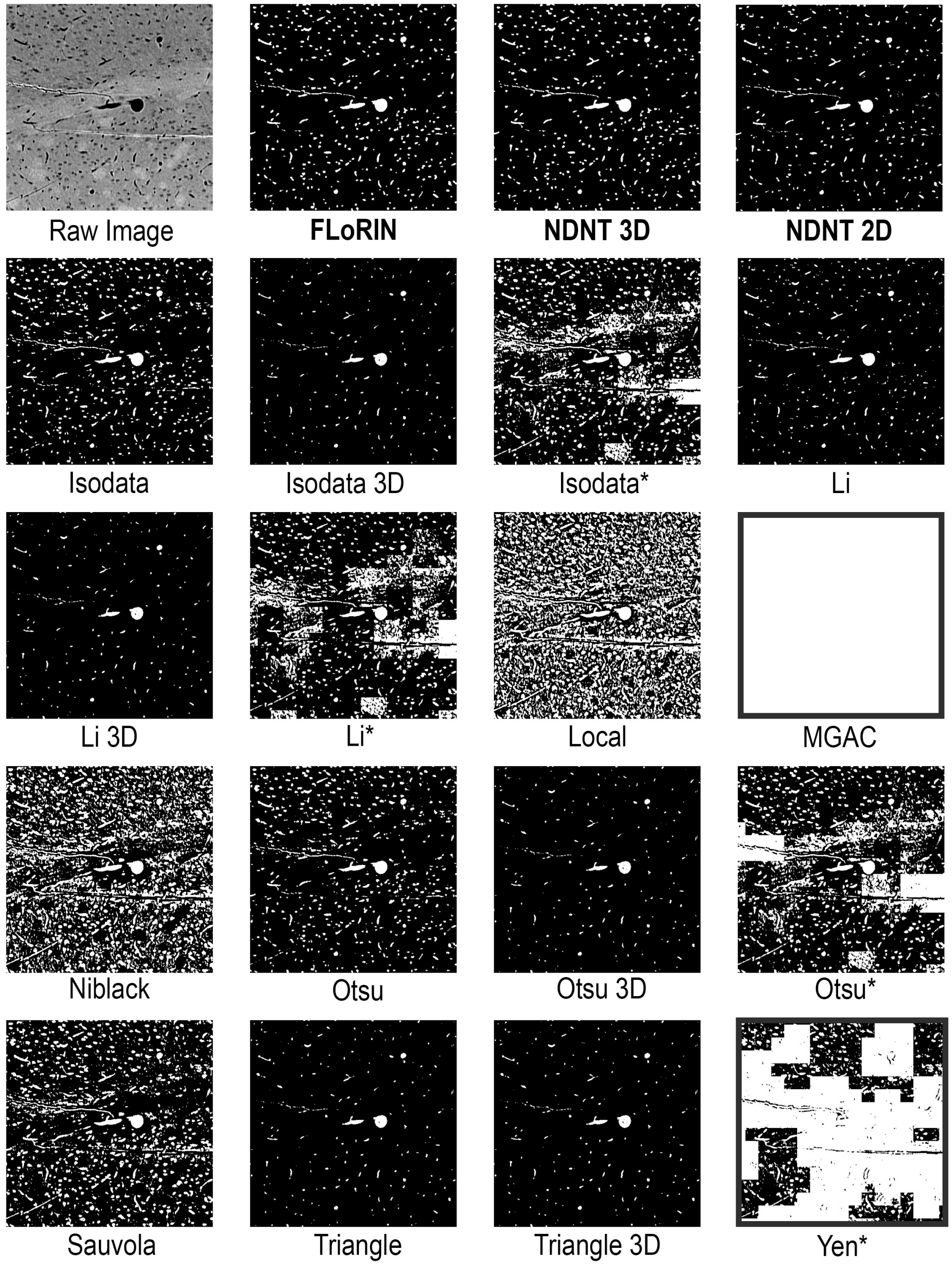

Supplementary Figure S 4. One of the ALRB dataset images from the μCT X-ray modality and the segmentation results of various methods. The first row shows the result processed by the FLoRIN pipeline, and results of thresholding with NDNT 2D and 3D. Methods labeled with asterisks were applied over 3D blocks of the volume, which in extreme cases resulted in the white "tiles" in some of the images. Qualitatively, the Isodata[4] and Otsu[5] segmentations are similar to the NDNT results, however this does not necessarily hold true for other datasets. The remaining methods either severely over- or under-segment the volume.

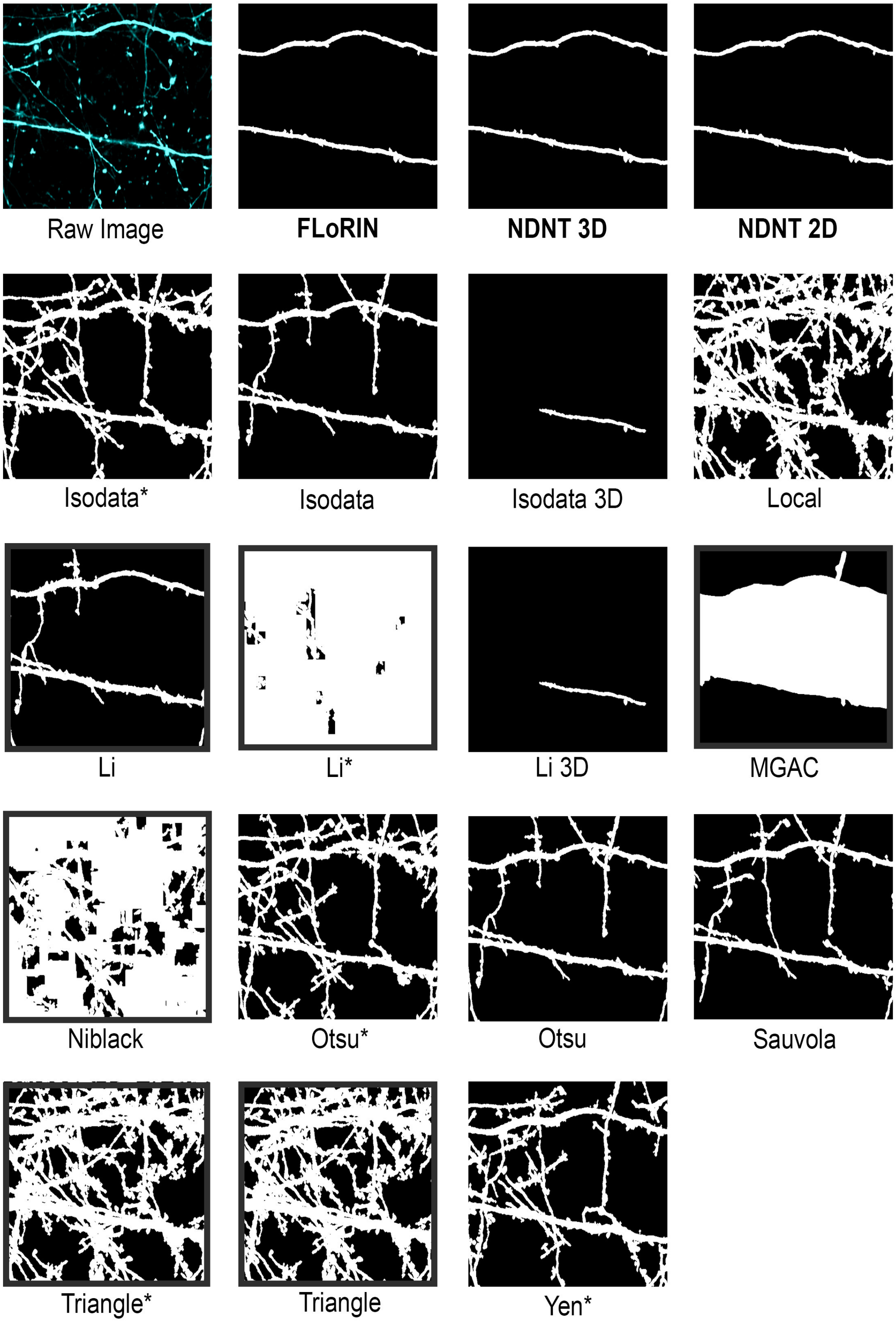

Supplementary Figure S 5. One of the SCoRE dataset images and the axon segmentation results of various methods. The first row shows the result processed by FLoRIN pipeline, and results of thresholding with NDNT 3D and 2D. All three of the first row segmentations correctly captured the two myelinated axons of interest without including any background axons. The Isodata[4], Li[8], and Otsu[5] results contain the fewest merge errors, of the additional methods evaluated, however all three segmented a perpendicular axon that connects the two axons of interest, which is incorrect. Other methods were consistently confounded by background axons.

References

1. Aspert, N., Santa-Cruz, D. & Ebrahimi, T. Mesh: Measuring errors between surfaces using the hausdorff distance. In *Multimedia and Expo, 2002. ICME'02. Proceedings. 2002 IEEE International Conference on*, vol. 1, 705–708 (IEEE, 2002).
2. Ronneberger, O., Fischer, P. & Brox, T. U-net: Convolutional networks for biomedical image segmentation. In *International Conference on Medical Image Computing and Computer-Assisted Intervention*, 234–241 (Springer, 2015).
3. Zack, G. W., Rogers, W. E. & Latt, S. A. Automatic measurement of sister chromatid exchange frequency. , Automatic measurement of sister chromatid exchange frequency. *J. Histochem. & Cytochem.* **25**, 741–753 (1977). URL https://doi.org/10.1177/25.7.70454. DOI 10.1177/25.7.70454.
4. Ridler, T. & Calvard, S. Picture Thresholding Using an Iterative Selection Method. *IEEE Transactions on Syst. Man, Cybern.* **8**, 630–632 (1978). DOI 10.1109/TSMC.1978.4310039.
5. Otsu, N. A Threshold Selection Method from Gray-Level Histograms. *IEEE Transactions on Syst. Man, Cybern.* **9**, 62–66 (1979). DOI 10.1109/TSMC.1979.4310076.
6. Niblack, W. *An Introduction to Digital Image Processing* (Strandberg Publishing Company, Birkeroed, Denmark, Denmark, 1985).
7. Yen, J.-C., Chang, F.-J. & Chang, S. A new criterion for automatic multilevel thresholding. *IEEE Transactions on Image Process.* **4**, 370–378 (1995). DOI 10.1109/83.366472.
8. Li, C. H. & Tam, P. K. S. An iterative algorithm for minimum cross entropy thresholding. *Pattern Recognit. Lett.* **19**, 771–776 (1998). URL http://www.sciencedirect.com/science/article/pii/S0167865598000579. DOI 10.1016/S0167-8655(98)00057-9.
9. Sauvola, J. & Pietikäinen, M. Adaptive document image binarization. *Pattern Recognit.* **33**, 225–236 (2000). URL http://www.sciencedirect.com/science/article/pii/S0031320399000552. DOI 10.1016/S0031-3203(99)00055-2.
10. Itseez. *The OpenCV Reference Manual*, 2.4.9.0 edn. (2014).
11. Márquez-Neila, P., Baumela, L. & Alvarez, L. A Morphological Approach to Curvature-Based Evolution of Curves and Surfaces. *IEEE Transactions on Pattern Analysis Mach. Intell.* **36**, 2–17 (2014). DOI 10.1109/TPAMI.2013.106.
12. Joesch, M. *et al.* Reconstruction of genetically identified neurons imaged by serial-section electron microscopy. *eLife* **5**, e15015 (2016).
